# Exploring agency and entrainment in joint music-making through the reported experiences of students and teachers

**DOI:** 10.3389/fpsyg.2022.964286

**Published:** 2022-11-24

**Authors:** Eveliina Stolp, Josephine Moate, Suvi Saarikallio, Eija Pakarinen, Marja-Kristiina Lerkkanen

**Affiliations:** ^1^Centre of Excellence in Music, Mind, Body and Brain, University of Jyväskylä, Jyväskylä, Finland; ^2^Department of Teacher Education, University of Jyväskylä, Jyväskylä, Finland; ^3^Department of Music, Art and Culture Studies, University of Jyväskylä, Jyväskylä, Finland

**Keywords:** agency, entrainment, joint music-making, music education, teachers, students, intersubjectivity

## Abstract

This qualitative interview-based study draws on the reported experiences of students and teachers to explore how agency and entrainment resource and constrain each other in joint music-making. The participants were 23 students of Grades 6 and 11 music teachers from different primary schools. The qualitative content analysis of the 11 student pair interviews and 11 one-to-one teacher interviews indicated that experiences of music-related interpersonal entrainment intertwine with different dimensions of agency. In the analysis, four themes were identified as follows: presence, belonging, safety, and continuity. These findings provide insights into the relationship between agency and entrainment in classroom-based joint music-making and provide a novel lens through which to examine the complementary experiences of students and teachers. This study builds bridges between the concepts of agency and entrainment in the context of music education, offering theoretical clarification as to how and why joint music-making can be considered an intersubjective activity that fosters group cohesion and social interaction. The findings further present a view of the constitutive nature of the relationship among agency, entrainment, and intersubjectivity in joint music-making. The findings offer educators concrete grounds for using joint music-making as a platform for an agency.

## Introduction

A number of studies show how singing together promotes one’s wellbeing and has a significant impact on various factors of social cohesion (Pearce et al., 2015; Salminen, 2020). Many individuals find joint musical action profoundly and emotionally satisfying—being part of socially constructed music-making results in positive experiences and emotions (Pearce et al., 2015). Feeling, hearing, and sharing togetherness through music are experiences that many of us can identify with. These moments and encounters with music are highly affective, relational in nature, and based on communication that goes beyond words (Trondalen, 2016). When individuals come together to dance or to make music, their joint action is characterized by entrainment, that is, mutual sharing and complex rhythmic timing (Phillips-Silver and Keller, 2012). However, little is known regarding how entrainment benefits from and contributes to student agency, that is, students’ willingness, capacity, and interest to act (Skinnari, 2014; Rajala et al., 2016) in music education. Thus, this study explores how agency and entrainment resource and constrain each other in joint music-making. As a qualitative study, this research focuses on the social nature of music and the potential of music to help children move beyond a sense of individual self to a shared sense of “us.”

Entrainment in sociomusical contexts is used to describe and characterize in-time interactive synchronization between two or multiple participants through music (Phillips-Silver and Keller, 2012; Cross, 2014). Although entrainment is often regarded as a spontaneous neurophysiological process influencing cognition, the concept of interpersonal entrainment, defined as the interaction and coordination between human beings mediated by sound and movement (Clayton et al., 2020), highlights entrainment’s social nature. Moreover, the social nature of entrainment is highlighted in different levels of musical entrainment. One level is intra-individual entrainment which involves the perception of metrical structures in music and the coordination of actions. A second entails inter-individual and intra-group entrainments, which pertain to coordinating actions between individuals in a group, while a third is an inter-group entrainment which includes coordination between different groups (Clayton, 2012). These different levels emphasize the embedded nature of social context, conscious awareness, the experience of being in time, and the contribution to sociality (Kim et al., 2019). They also suggest that entrainment can also be explored and examined at different levels through different approaches (Clayton et al., 2020).

Although interpersonal entrainment is affected by social considerations, such as the participants of the group, their interactions and the environment, resources available, the knowledge the participants hold, and how their internal representations enable them to participate (Clayton et al., 2020), these aspects of interpersonal entrainment have received little attention to date. Recognizing that interpersonal entrainment is characterized by intentionality, active participation, and mutual sharing (Trondalen, 2016; Clayton et al., 2020) creates an opportunity to explore joint music-making from a qualitative rather than quantitative perspective. Our study took place in Finland, where joint music-making is one of the core curriculum’s central methods used in music education in basic education (EDUFI, 2016). Joint music-making involves every student in the classroom playing together as a group, despite their background or perceived skill. In joint music-making, students are encouraged to try different instruments, body percussion, a range of musical genres, and various ways of participating in promoting their musical and social skills. The goal is to teach and bring the whole class of students together to play at the same time and to experience interpersonal entrainment through musical activity. While including joint music-making as part of the curriculum is an opportunity for students to develop the ability to be entrained with others and to be in time (in sync) with others (Cross, 2014), it cannot be assumed that students will automatically enter into joint music-making. Arguably, as in other areas of the curriculum, joint music-making requires skills that develop through practice and the willingness to act and participate (e.g., Skinnari, 2014; Rajala et al., 2016). In other words, joint music-making is in part resourced by the agency of students.

Student agency has become a central concept in recent educational research (Niemi et al., 2015; Kangas et al., 2017; Vaughn, 2020). It is widely acknowledged that supporting the agency of children advances the development of a responsible society by increasing their competence and motivation to encounter changes in life, advancing equity, and encouraging them to take responsible actions that will make a difference (Vaughn, 2020). In this study, we use a sociocultural conceptualization of agency as a dynamic process interdependent on the individual and social aspects where a subject actively chooses, acts, and reconstructs their worlds (Eteläpelto et al., 2013), thus complementing the social considerations of entrainment. Furthermore, student agency is fostered through *a student’s experience of having access to or being empowered to act through personal, relational, and participatory resources, which allow him/her to engage in purposeful, intentional, and meaningful action and learning in study contexts* (Jääskelä et al., 2020, p. 2).

However, to date, a limited amount of research has shed light on student agency in music education, where interpersonal entrainment plays a central role in the joint music-making of everyday classroom settings. Furthermore, little is known about children’s or teachers’ experiences in this process. Thus, the current study contributes to the discussion about the agency not only by exploring the relationship between entrainment and agency but also by investigating both student and teacher experiences of the process to gain an understanding of joint music-making as a forum for the agency. The aim of this study is to explore children’s and teachers’ experiences of joint music-making and to ascertain whether and how student agency contributes to joint music-making and whether entrainment supports the agency of students. This is particularly important when we are developing new pedagogical practices and transforming music education practices to support human agency and to understand the social aspects of interpersonal entrainment.

## Subject-centered sociocultural approach to agency

In this study, we drew upon a subject-centered sociocultural approach to conceptualize agency (Eteläpelto et al., 2013). Previous studies on student agency have indicated how both teachers’ and students’ active participation in education leads to effective learning, and how teaching practices can increase the agency of the students (Niemi et al., 2015; Vaughn, 2020). The subject-centered sociocultural approach (Eteläpelto et al., 2013) views individuals as feeling and willing agents who actively and continuously reconstruct their realities by choosing and considering what is worth pursuing within a complex interplay of individual, social, temporal, cultural, and material aspects of agency. This approach conceptualizes agency as a constantly evolving process over time in relation to social and material environments in terms of their constraints and resources (Priestley et al., 2015; Jääskelä et al., 2020). The work of Jääskelä et al. (2020) highlights how student agency in tertiary settings relates to certain *individual* resources, such as competence beliefs, self-efficacy, and intrinsic motivation, *relational* resources including emotional atmosphere, experiences of trust, support, and power relations, and finally, *participatory* resources referring to subjects’ experience of the opportunities for active participation, influencing, and making choices. Eteläpelto et al. (2013) argued that agency should not be analyzed by focusing on the events of action, as the agency can manifest itself eclectically. Rather, the subject’s interpretations, meanings, and purposes in the process of manifesting agency need to be considered, which can appear, for example, as resistance to or purposeful maintenance of existing practices.

A subject-centered sociocultural approach connects with the socio-cognitive perspective of Bandura (1986, 1999) in that perceived self-efficacy is the most central foundation of human agency. From a socio-cognitive perspective, the agency is approached by emphasizing the individual capacity to influence and produce the desired effects by one’s actions, where the belief in that capacity is intrinsic to the evaluation of goal setting. While a socio-cognitive perspective (Bandura, 1986, 1999) usefully highlights the individual experience of determination, self-esteem, and competence, which play an influential role in motivation, the focus on the individual tends to bypass the significant role of others and environmental conditions in the realization of agency. Nevertheless, the subject-centered sociocultural approach acknowledges the processes by which subjects construct and practice their agency and focuses on how the subject learns through these processes (Eteläpelto et al., 2013). Furthermore, it is acknowledged that agency cannot be considered as a power that an individual possesses but, conversely, as a dynamic process where the agency can be achieved through engaging with unique temporal–relational contexts and environments (Emirbayer and Mische, 1998; Biesta and Tedder, 2007). Thus, the agency can only be realized in the present moment; it cannot be separated from the involvement of the past and the future, or the available resources or structural factors provided by the environment (Biesta and Tedder, 2007; Eteläpelto et al., 2013; Priestley et al., 2015).

An increasing amount of research acknowledges the role of emotions in enhancing or hindering the actions and agency of an individual (Eteläpelto et al., 2013; Slaby and Wüschner, 2014; Ruud, 2020). In particular, tertiary educational research has indicated how an emotionally safe climate in learning situations facilitates student agency (Ruohotie-Lyhty and Moate, 2016; Juutilainen et al., 2018; Jääskelä et al., 2020). As emotions are the primary indicators of what we regard as potential doing or potential happening (Slaby and Wüschner, 2014), we believe emotions are a significant prerequisite in the process of agency as they demonstrate resistance or active participation in social action. The research on students’ agency recognizes the presence and range of emotions children experience and use to inform and understand their agentic action and participation (Kirby, 2020; Mameli et al., 2021). Students’ agency is not just carrying out the instructions of teachers, but students’ actions alter the shared space of the classroom, the possibilities of teaching and learning activities, and their experiences (Kirby, 2020). Arguably, this creates a foundation for future action or inaction (Emirbayer and Mische, 1998).

### Approaching agency through interpersonal entrainment and intersubjectivity in joint musical action

In addition to student agency, musical agency, as an important part of music education practices, has been acknowledged in research (Karlsen, 2011; Juntunen et al., 2014; Saarikallio, 2019; Ruud, 2020). Musical agency refers to both individual and collective ways of interacting with music (Karlsen, 2011), becoming aware of one’s personal, relational, and material resources (Ruud, 2020), and being a resource for development, empowerment, and identity construction (Saarikallio, 2019). For example, in Karlsen’s (2011) work, the individual dimension of musical agency includes aspects of how one can use music to extend one’s position in the world and to construct oneself in relation to others, such as perceiving and playing music, shaping self-identity, and using music for self-regulation. The collective dimension of musical agency (Karlsen, 2011) includes aspects of how collectively, through music, it is possible to explore social relationships, regulate and structure social encounters, and affirm and establish collective identity.

Musical entrainment, to a degree, is present from the early stages of a life span (Winkler et al., 2009; Zentner and Eerola, 2010), and it continues to develop and change over time. Entrainment for a primary school student, for example, is not as precise as for professional musicians (Clayton et al., 2020), which suggests that entrainment is something that can be partly learned and developed. Entrainment has been widely studied in music education and music therapy contexts, as it has been regarded as a significant factor in uniting humans throughout their life span. Rhythmic entrainment has been argued to result in prosocial behavior and foster the social competence of children (Kirschner and Tomasello, 2010; Ilari et al., 2018). Furthermore, in the work of Mogan et al. (2017), synchronous actions were found to affect prosocial behavior, perceived social bonding, social cognition, and positive affect. As previous studies have indicated that entrainment contributes to prosocial behavior and social cohesion, they justify entrainment being further studied from a social perspective. Thus, in our research, we focus on the social dimension of interpersonal entrainment in a joint music-making context. From the social theory perspective, musically entrained behavior, like music and dance, connects profoundly to human sociability (Clayton et al., 2020), which plays a central role in the conceptualization of a subject-centered sociocultural approach to agency (Eteläpelto et al., 2013).

Research suggests that synchrony drives prosocial effects, such as cooperative behavior and empathy, through affective mechanisms (Mogan et al., 2017). Also, Launay et al. (2016) have argued how synchrony, active engagement in musical activities, and moving together influence social bonding by emphasizing the role of neurohormones, such as oxytocin and endorphins. Furthermore, Trost et al.’s (2017) work illustrates why entrainment could be experienced as a desirable and pleasant state, as they found that a level of mostly positive and arousing emotions is induced through perceptual, autonomic physiological, motor, and social entrainment. However, although the mechanisms between entrainment and emotional experiences remain unclear (Clayton et al., 2020), the connection between affective entrainment of sharing affective states between individuals in joint music-making and social bonding has been strongly suggested (Phillips-Silver and Keller, 2012). Thus, entrainment in joint music-making should be understood through social and interpersonal synchronization with complex affective experiences, in addition to synchronizing with the beat alone (Phillips-Silver and Keller, 2012; Clayton et al., 2020).

The work of Clayton et al. (2020) proposes a new model of interpersonal entrainment that compares two separate components, synchronization and coordination within musical contexts, in terms of their role in culturally shared knowledge and the connection between entrainment and social processes. They argue that there is a need to study this phenomenon in relation to other social processes in order to increase our understanding because, alongside aspects of evolution, development, psychology, and neurophysiology, there are also social and cultural dimensions that have a clear impact on interpersonal entrainment. They describe how social, material, environmental, and cultural aspects affect interpersonal entrainment, and how knowledge, in other words, being able to plan and anticipate, plays an important part in the process of interpersonal entrainment. As participation and engagement are necessary for the realization of interpersonal entrainment in joint music-making, it requires highly agentic action and will from an individual to take part in social music-making. In other words, entrainment in collaborative musical action does not just happen; rather, individuals have to want, be able to, and actually act for entrainment to be possible. Repetition and loops open participatory musical forms and create a sense of safety (Turino, 2008), whereas the dense musical texture hides “mistakes” made by individuals and eases the contribution at different skill levels (Ruud, 2020). Ruud (2020) suggests that the participatory aspect of making music together emphasizes contribution and democracy among participants instead of musical skills. Possibly, the music itself creates a safe environment, thus resourcing opportunities for agentic action by students.

As in the work of Phillips-Silver and Keller (2012) and Trondalen (2016) points to the significance of affective experiences and intersubjectivity (Stern, 2004) when seeking an understanding of the relational nature of joint music-making in the here and now. According to Trondalen (2016), intersubjectivity, at the practical level, encompasses awareness and the exchange of affects in time, as well as experiencing each other through the body, affect, and such experiences of “I feel that you feel that I feel.” Microprocesses, such as movements, facial expressions, timing, and intensity of moment to moment, are unspoken and relational, and they form the basis for subjectivity, togetherness, possibilities of action, and language itself in the intersubjective process among participants (Stern, 2004; Trondalen, 2016). Similarly, Vass (2019) argues how musical encounters, embodiment, and mutual resonance open the way to dialogical space that is a prerequisite for intersubjectivity, the nature of which reaches beyond the verbal world and intellectually constituted thought. Furthermore, Vass (2019) highlights students’ embodied agency in the joint musical activity that opens a unique way to the center of learning through the embodied experience of knowing and relating. These aspects of intersubjectivity in joint music-making elucidate the multimodal environment and the available and constraining resources for the construction and development of agency, as likewise, intersubjectivity is regarded as a key quality of agency (Damşa et al., 2010; Vass and Littleton, 2010).

Becoming entrained points to conscious relational experiences and the non-verbal interactive processes that are inseparable aspects of joint music-making (Trondalen, 2016). As Cross (2014) highlights, interaction in music is something other than interaction in language because music can provide a space to sense others in which the participants can align their attitudes and motivations with those of others. Similarly, Trondalen (2016) emphasizes that through verbal conversation, it is possible to describe the meaning of musical experiences, but it can never replace the experience of meaning at the non-verbal level. These experiences also shed light on *implicit relational knowing*, that is, one’s feeling of oneself and others in the here and now when making music together. Implicit relational knowing emphasizes one’s feeling of how to do things with others in a musical presence, as its essence lies in the attunement of affect and involves joint intersubjective recognition. This expands an individual’s state of consciousness and increases the opportunities to create new ways of being together (Trondalen, 2016). Thus, in this study, joint music-making is considered to be a holistic, highly interactive, embodied, and affective process among individuals through music, which creates a platform for subjective experiences and agentic action. While measurements provide insight into physiological changes and alignments, quantitative research provides little insight into the conscious activity of participants. Interpersonal entrainment no doubt involves spontaneous neurophysiological processes, but more understanding is needed about the intentional participation of individuals as a part of entrainment as well as the individual, social, and cultural processes that influence interpersonal synchronization and coordination in music.

## Aim of the study

In the present study, we explored the relationship between entrainment and agency through the reported experiences of students and teachers in joint music-making. The following research questions focused more specifically on:

(1) What characteristics of agency are present in the reported experiences of students and teachers in joint music-making?
(2) In what ways do agency and entrainment resource and constrain each other in joint music-making?

## Methodology

This qualitative interview-based study draws on the reported experiences of 11 teachers and 23 students. The findings reported here belong to a larger study examining different aspects of music education in Finnish primary education. The overall dataset comprises 11 teacher interviews and 11 student pair interviews. The first study focused on teacher beliefs about student agency in whole-class playing, in which an abductive approach was used to identify and examine the manifestations of student agency and musical agency (Stolp et al., 2022a). An abductive approach (Timmermans and Tavory, 2012) was chosen as it allows the interplay between data and existing theories as well as the extension of theoretical perspectives through the creative process. The abductive analysis was double-coded by two coders for teacher interviews, and intercoder reliability was 93.75%. The findings highlight the significance of student agency in joint music-making from the teacher’s perspective. A second study examined students’ experiences of whole-class music playing (Stolp et al., 2022b). A complementary insight from the two initial studies was the significance of interpersonal entrainment in relation to agentic activity and joint music-making, described as, for example, *“The most significant thing in joint music-making is that through music, through musical experience, and with the help of music, you become part of something bigger than yourself and you have a special role in the group. That you kind of disappear and you become part of an entity. Once you get the experience that you are part of the wholeness of the sound, with the sound you produce, and once the wholeness sounds very good, you want to do it again”* (Teacher 10) and *“Everyone is focused and concentrated on the same song. The best thing is that we have a good rhythm, and everyone is participating actively and seriously”* (Student 10). The study reported here brings the teacher and student interview datasets together to specifically explore the interpersonal entrainment resources and benefits from student agency in joint music-making.

### Participants

The individual teacher and student pair interviews were conducted when strict guidelines were in place due to the COVID pandemic. While the pandemic significantly limited access to research participants, the participants in this study include 11 music teachers and a class of sixth-grade students (23 students, 12–13 years old). In Finnish schools, there is usually only one specialist music teacher who teaches music to nearly all pupils in one comprehensive school, although a willing and qualified class teacher can also teach music. The teacher participants from various schools in Central Finland were recruited through school email or social media. Student participants were recruited *via* one of the music teacher participants. The selection of Grade 6 students was guided by the timetables of the school and the interviewer (first author) as well as the willingness and ability of the students to participate in the pair interviews.

With regard to increasing the diversity of the teacher participants, we sought variation in their educational background, the schools they taught in, work experience, and age. All of the teacher participants taught music in Grades 1–6 in Finnish basic education. Three of them were qualified as music teachers and three as class teachers, and four of them had the qualification of both music and class teachers. The teacher participants’ backgrounds, including their gender, age, education, and work experience, are presented in Table 1. Due to the pandemic, it was not possible to recruit more student participants; nevertheless, the class of 12–13 years old included 11 male students and 12 female students with a variety of musical backgrounds, ranging from enthusiastic hobbyists to those who only regarded music as a compulsory school subject.

**TABLE 1:** Backgrounds of the participants in the study.

Participants	Number	Age	Male/Female	Work experience (teachers)	Education (teachers)
Teachers	11	28–55	2/9	2–25 years	Class teacher (*n* = 3) Music teacher (*n* = 4) Class + Music teacher (*n* = 3)
Students (Grade 6)	23	12–13	11/12		

### Ethics and procedure

Before contacting the student participants, we contacted the ethics committee of the university for the evaluation of the need for an ethical review to be carried out for our study. However, consent from student participants’ guardians was enough to comply with the guidelines of the Finnish National Board on Research Integrity (TENK, 2019). No ethical review was required. Consent forms were collected from all teacher participants. Permission for the study was obtained from the municipal school authorities and the principals of the schools involved, and teacher participation was voluntary. Before data collection, the teacher participants, as well as the student participants and their guardians, were informed by the first author about the purpose of the study, methods, and ethical commitments, after which oral consent was given by the students, and written consents were signed by their guardians and teachers. Prior to the student interviews, the first author also visited the students’ class to give them information, such as explaining that the questions would be about their experiences of joint music-making and to reassure them that there would be no need to prepare for the interviews. The students were also told to just be honest when answering the questions and that they would be able to ask questions. Teacher participants were interviewed in October 2020, and the students were interviewed during normal school hours on the school premises in April 2021.

### Student interviews

Semi-structured interviews (Tracy, 2013) were conducted by the first author and guided by the subject-centered sociocultural approach to student agency (Jääskelä et al., 2020) and musical agency (Karlsen, 2011). The class teacher divided the student participants into pairs and one triad prior to the interviews by allowing the students to freely choose their pairs. The questions were designed so that the students could easily answer descriptively and concretely but also be able to share their experiences and perspectives of joint music-making. The questions concerned the students’ relationships with music and their actions in the process of joint music-making. For example, a typical question was as follows: *When you begin to play a song with your class, what are the things encouraging you to participate?* As theories (Karlsen, 2011; Jääskelä et al., 2020) indicate, there are individual, relational, participatory, and musical resources of agency that either foster or limit agency; our questions were formed in a way that participants could give us examples of what affects agency in joint music-making. All the questions used in the interviews are presented in Appendix 1. The interviews (lasting from 6 to 15 min) were conducted as pair interviews (with one triad) in a small room during the lessons. It was noticeable during the interviews that when one student shared their reflections, their pair often built on this reported experience by sharing something similar or using this as a contrast. The interviews were recorded, transcribed, and anonymized. The final dataset included 103 pages of transcribed text (double-spaced, 12-point font).

### Teacher interviews

Interviews were conducted remotely on a one-to-one basis by the first author *via* Zoom or Microsoft Teams. Before the interviews, the teachers were asked to complete a short questionnaire on their background information, including their gender, age, education, and work experience. This information was used only to ensure the diversity of the sample and was not used in the analysis. The semi-structured interviews (Tracy, 2013) were guided by a subject-centered sociocultural approach to student agency (Jääskelä et al., 2020) and musical agency (Karlsen, 2011). The questions allowed teachers to reflect on their own experiences and meanings of being part of joint musical action, and space was provided for them to share their experiences and perspectives of joint music-making playing as teachers. An example of an interview question is the following*: In your opinion, what affects how a whole group of students starts to play together, and how is the joint music-making playing situation built?* All the interview questions are presented in Appendix 2. The average length of interviews varied from 30 to 60 min, and the recorded interviews were transcribed and anonymized. The final dataset included 260 pages of transcribed text (double-spaced, 12-pt. font).

### Data analysis

**FIGURE 1:**
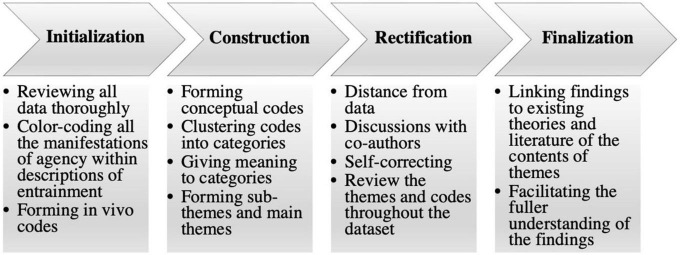
The process of theme development after Vaismoradi et al. (2016).

The data were analyzed within a qualitative content analysis framework (Vaismoradi et al., 2016). While our interest in the agency in music education informed the data collection process, our four stages of analysis included both abductive (first stage) and inductive (second, third, and fourth stages) approaches to be as open as possible to the themes raised by the student and teacher participants. In the analysis, we adapted Vaismoradi et al.’s (2016) theory of the theme development process, as shown in Figure 1. The goal of the qualitative content analysis was to identify the themes that reflected the experiences of the participants regarding agency during entrainment in joint musical action.

**FIGURE 2:**
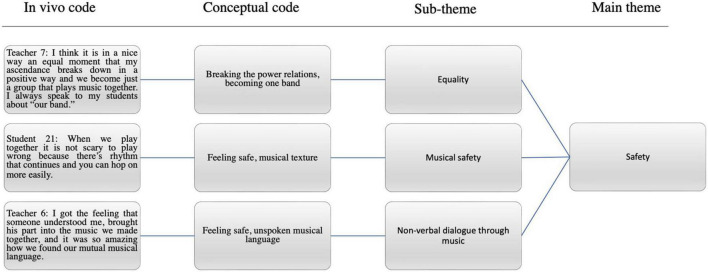
Phases of coding and theme development.

Teachers’ interviews were double-coded by two independent coders for calculating intercoder reliability, which was 93.75%. Since the first author ran the double-coding process of the teachers’ interviews, she was also responsible for the coding of the student interviews, which were not double-coded. However, the third stage of the data analysis process included going through the data analysis with the co-authors and discussing the codes and themes as there were also some unclear codes that were placed together under certain themes to ensure the integrity of the analysis. The overall analysis consisted of four stages. The first stage, which was abductive, was to become familiarized with the data through careful rereading, identify the manifestations of agency based on the individual, participatory, and relational dimensions of student agency in the descriptions of experienced entrainment in the transcribed text, and utilize them as *in vivo* codes. The second stage was to sort the *in vivo* codes, based on their similarities, into conceptual codes and further sort these codes into potential themes (see Figure 2). After 11 student pair interviews and 11 one-to-one teacher interviews, data saturation was reached, meaning that the data were collected until nothing new was apparent (Tracy, 2013). Third, after distancing, discussions with co-authors, and self-correcting, potential themes were reviewed to ensure that the themes were representative of meanings that arose in the data. The final stage in the analysis was to examine the findings in relation to existing theories and literature to facilitate a deeper understanding of the findings.

## Findings

The findings of the study are presented through four themes identified in the analysis: presence, belonging, safety, and continuity, which opens a unique pathway to view the relationship between experiences of agency and subjects’ experiences of entrainment and the resources and constraints involved. Each theme is presented in its own sections.

### Presence

When discussing playing together as a group, both student and teacher participants emphasized in many ways the meaning of being present in the moment, here and now. In a way, presence crystallizes the collective, focused moment where individuals become visible and aware of not only their environment and themselves but also others, as Teachers 11 and 9 described:

Teacher 11: It starts from the moment, while we play, when I get to engage every single student in that very moment by saying that “you are here now, I see you, and you are part of this thing that we do together” and “you, as yourself, are part of this and it is enough” and “how cool [it] is that you are here and part of our band!”

Teacher 9: It is like living the music in the moment together. It means that our awareness and our attention is focused on the mutual thing that we are doing together. It requires that our attention cannot be focused on anything else at that moment. It is, like, here and now, meaning that it is something you cannot do remotely tomorrow or alone during the break.

Students also described entrainment in their experiences of joint music-making as having a “good rhythm,” and as intensive moments of collective concentration and focus, which they seemed to be willing to aim for and maintain. Experiences of entrainment made them feel positive emotions when succeeding or, on the contrary, disappointment if the level of engagement of peers was low, as indicated in the following extracts:

Student 1: When everyone concentrates and participates and succeeds it makes you very happy.

Student 10: Everyone is focused and concentrated on the same song. The best thing is that we have a good rhythm, and everyone is participating actively and seriously.

Student 8: I think it is because we are playing in the same rhythm and we all play the same thing, and when everyone concentrates, then everyone stays in the same rhythm.

Student 12: The attitude of the others, like how they come to participate and if everyone concentrates. If they don’t, you can’t concentrate either.

These extracts indicate how experiences of entrainment, which is often related to an unconscious body synchronization process, connect not only with various aspects of musical agency, such as consciously perceiving a good rhythm that participants produce collectively by playing their instruments but also becoming aware of relational and individual aspects of agency, such as peers’ support, active participation, and motivation.

Interestingly, teacher participants provided insights into how presence in joint music-making is a strongly embodied experience, as teachers described how they became aware of themselves physically. Playing together was felt in their bodies as “resonance” (Teacher 9), “vibe” (Teacher 2), or how “the body just starts to go along” (Teacher 7). There were also comments in the teacher interviews about how the presence in music was experienced bodily by their students. Teacher 8 described how a student would refuse to play any instrument, but as the playing started, the student immediately started to physically “sulk in tempo.” Teacher 7 described how when there were not enough instruments for all students, those without instruments began swinging and moving to the music while others played.

The findings indicate how presence in joint music-making includes becoming aware of the embodied experience but also actively sensing and becoming aware of shared feelings that are present in the moment, as in the following comments from teachers and students:

Teacher 10: There is always shared emotional experience that takes shape in playing together, like enthusiasm for example. I can see the enjoyment from their faces.

Teacher 1: It was a memorable experience for all of us, me and the students, because we were so excited. We just went to play together during the breaks because we enjoyed it.

Teacher 6: It is a kind of emotional language, or a way to experience emotions together. You have to experience it and be part of that before you can give meanings to your experience.

Teacher 11: We [students and teacher] always stop at some point just to reflect on how we feel after we manage to play something together.

Student 9: The moments when everyone is able to concentrate and is happy in what they do are precious moments. It feels like we have a good team spirit in those moments.

The findings indicate how presence and the immediacy of the moment in joint music-making are elements that often defuse the resistance of a student and help them to focus. Teachers described how “a resistant student changes course quickly” (Teacher 11), “no one bothers to fool around” (Teacher 1), and ‘hyperactive and unfocused students just start playing without a problem” (Teacher 10) once the music and playing start.

The findings point to the active and intentional way of becoming aware of the moment with music, self, and others, engaging with the moment by concentrating and participating, and feeling the shared positive emotions that are taking shape through the collaborative act. These experiences connect with individual resources of agency, such as motivation and self-efficacy, through experiences of success and positive emotions, but also with relational resources, such as peer support, which intertwine with participation activity and a will to be part of an experience. Becoming entrained with others in these experiences leads to increased agentic participation, affect attunement, and joint focus in the very moment, which are central aspects in intersubjectivity and supply feedback to agency. However, the environmental constraints, such as peers having problems with concentration and the availability of instruments, during joint music-making have a significant impact on the participatory resources of agency, as to at what level it is possible to take part in the activity. It is, after all, that “music lives in time. It is a unique sound that we produce in this very unique moment, and this sound and moment will never come again” (Teacher 10).

### Belonging

The student and teacher participants provided insights into the belonging and sense of togetherness during joint music-making in both social and musical dimensions, a moment before students “disappear” into the entity and become a collective “one.” They described how, through music, they perceived themselves as being part of a musical and social entity, through which they experienced strong positive emotions, such as feelings of success or enthusiasm, and which further strengthened their agency such as their willing to actively take responsibility in entering or maintaining activity. These descriptions of musical experiences where one disappears and becomes part of an entity with the help of music, and being part of the wholeness of the sound which sounds good, are interpreted as experiences of entrainment. Teacher 10 described this:

The most significant thing in joint music-making is that through music, through musical experience, and with the help of music, you become part of something bigger than yourself and you have a special role in the group. That you kind of disappear and you become part of an entity. Once you get the experience that you are part of the wholeness of the sound, with the sound you produce, and once the wholeness sounds very good, you want to do it again.

Describing a special role in the entity and producing sound underlines the individual aspects of agency, such as active participation and competence, and the relational aspects, such as becoming collective “one” with and with the help of peers. By mentioning “you want to do it again” points to the individual aspect of agency where motivation, which is central to agency, seems to be fostered through the musical experience of entrainment. Moreover, there are both individual and collective aspects of musical agency present in this extract: actual playing, perceiving music and its elements, and collaborative musical action.

The theme of belonging was based on two sub-themes, the social and musical, which could partly be analytically separated but overlapped and constituted each other. In the interviews of students, these sub-themes were easier to separate, whereas, in teacher interviews, they were intermeshed in a way that made it almost impossible. The significant factor in all these experiences of belonging, in terms of the social and musical aspects, is that they awaken positive emotions that are closely linked to agency (Slaby and Wüschner, 2014) when considering and choosing what is worth pursuing or maintaining (Eteläpelto et al., 2013). In the student interviews, the social aspect was highlighted by Students 8 and 9, who stated how they “like to play with their class because you don’t have to be alone” and by Student 11:

The best thing is that we have a good team spirit when we play together.

There were a great number of comments by the students describing the musical togetherness, that is, actively perceiving the music and adapting their acts to collaborative musical action, which seemed to lead to satisfaction and experiences of success, as can be seen from the following comments:

Student 13: It sounds like real music when we all play together.

Student 4: The best thing in joint music-making playing is the fact how it sounds. When everyone plays instruments in the same tempo, it sounds really good.

Student 8: The best thing is what the end result sounds like if we succeed.

Student 17: We sound like a real band.

However, there were also comments from both students and teachers about how failing to maintain entrainment makes the sound unpleasant and causes feelings of failure, and how the entity is fragile and dependent on individuals and their efforts:

Student 5: If we all play together, then it might lose the same pace if everyone plays at a different tempo.

Student 4: If the class just messes around and does not succeed, then it just sounds stupid.

Teacher 9: It doesn’t work if we don’t listen to each other. If we are missing the joint tempo, it won’t work, and you can hear it and feel it in the same way you can hear and feel it when it works. And then the student gets the feedback right away if it works and whether he succeeded. That now we succeeded together. The feedback is immediate when something succeeds.

Teacher 7: Unfortunately, if the drummer, for example, can’t maintain the tempo, the whole group can get the experience of how the whole thing collapses.

In the teacher interviews, the social and musical aspects of belonging were more intertwined. Teachers shared their own meaningful experiences of joint music-making and moments of entrainment, as well as their experiences of their students playing together. These experiences shed light on the emotional aspect of both agency and entrainment, where the experience of entrainment opens the way to a sense of togetherness and which arouses positive emotions affecting and expanding participants’ beliefs about what they are capable of and willing to enter, as can be seen in the following comments:

Teacher 4: To me, the significance is in making something big together. Something much more than my own part alone. That together you can do more than you can alone. It is just the best thing being part of the great sound machine. Like it is such an amazing feeling if I play this, and you play that, and the entity sounds so good.

Teacher 2: That you feel you can have an impact on the joint sound that is produced together, realize your own part in that entity and how your part influences the whole. That you hear what the others are doing and perceive yourself as a part of an entity. Those are rewarding and motivating experiences.

Teacher 3: It was super nice when the class got the song to sound good together! When it starts to sound good, whether it was sung or played, it is a totally different thing to experience the moment of succeeding as a group than alone. I asked them, “Did you all notice?” and they were gasping, “It sounds ridiculously good!”

Teacher 7: Maybe the feeling when you notice that this is a shared experience of succeeding, that together we got this done and we sound amazing. We always toot our own horn if we succeed.

These extracts indicate how the moments of success are perceived through the quality of entrainment and reached through active participation, enactment of individual competence, and are both responding to and becoming aware of the support and emotions of peers and the teacher. In other words, the agency is needed for entering, perceiving, and becoming aware of the whole experience of entrainment, and it further facilitates the motivation and self-efficacy of an individual.

An interesting aspect of belonging was highlighted in one teacher’s comment that the sense of belonging in joint music-making can be so strong that it, in a way, can overcome a student’s insecurity of being competent through the experience and facilitate the self-efficacy of an individual, which is one characteristic of individual resources of agency:

Teacher 3: It was incredible because there was this student who could not play all the chords, but he did not care, did not toot his own horn, but instead played those chords he could. The entity did sound so nice that he was engaged and felt being fully part of that.

These extracts reveal how the increased motivation, sense of self-efficacy, and being capable of having an influence, not only individually but also collectively, comes out of the enactment of belonging, which requires active participation, willingness, and taking responsibility as an individual. These extracts also provide insights into the experiences of entrainment, as perceiving the musical structures and coordinating actions that connect with the experiences of togetherness and extend the relational resources of agency, such as the sense of safety and peer support. These experiences outlined here depict the resourcing and constraining relationship between agency and entrainment. They show how the ongoing social and musical interaction and the perception of oneself as being part of a larger entity are followed by positive emotional and musical feedback, which, in turn, contributes to motivation and agentic participation.

### Safety

Safety as a characteristic of joint music-making in the experiences of students and teachers relates to the interactional, musical, and relational aspects from a subject’s point of view that construct and shape the social environment through musical experiences. Joint music-making connects to an individual’s sense of safety, which is an important relational resource in facilitating the self-efficacy of an individual. Safety in joint music-making occurs through social interaction, such as listening, being responsive, respecting others, adjusting to them, and taking them and the responsibility for the collective into account. Students and teachers provided insights into the experiences of entrainment of how music itself creates a safe place to become part of something more because once the rhythm and dense musical texture go on, it is safe to make mistakes, as noted in the following comments from students and a teacher:

Student 21: When we play together, it is not scary to play wrong because there’s rhythm that continues and you can hop on more easily.

Student 20: You don’t have to be scared if you say lyrics wrong or play a wrong chord. You are not alone because we are together in it.

Teacher 11: I feel very safe and nice when together with people I do the same thing at the same time.

Experiences of teachers also point to the concept of equality, which is one central aspect of relational resources of agency, as differences in skill levels blend into the music and as the power relations break down as a result:

Teacher 6: Everyone plays the same. so there is nothing like someone being way better than anyone else *per se*.

Teacher 7: I think it is in a nice way, an equal moment, that my ascendance breaks down in a positive way, and we become just a group that plays music together. I always speak to my students about “our band”.

Teachers emphasized the meaning of social skills during joint musical action, such as the ability to “listen to each other,” “respect peers,” “give space to others,” “act responsibly,” “adjust,” and “be responsive.” In joint music-making, these interactions are non-verbal and enacted through music, which creates a platform for a multimodal dialog to unfold, as expressed in the following extract:

Teacher 11: In my opinion, successful joint music-making playing is an indication of the ability to listen to others and take others into account. I can demonstrate to my student that when he did this in music, he was part of the group, and that he respected his classmates by doing his role, and nothing else, and how, by doing so, he gave the space for others to do their thing.

Teacher 2 described how experiencing playing together is “like reading someone else’s thoughts in a different way than you do while speaking,” and Teacher 10 noted how “when we play together, we say much more than we can while speaking.” In other words, the experience of joint music-making and entrainment gives rise to intersubjectivity, increasing a sense of safety and mutual trust through social dialog that goes beyond verbal understanding. These experiences arouse positive emotions and seem to establish the sense of unity between people, as one teacher described:

Teacher 6: I got the feeling that someone understood me, brought his part into the music we made together, and it was so amazing how we found our mutual musical language.

Interestingly, this non-verbal social dialog might be one aspect that enables resolving tensions among a class that struggles with bullying and social fear. In joint musical action, the aspect of safety in the experiences of entrainment provides a group of students a chance to explore their mutual relationships and to affirm their unstable relational balance at an abstract level of social interaction. Another teacher shared an experience of classes with social tension that affected how they could (or could not) play together, and once the teacher had managed to get them to play together, there was an ease of doing and a will to keep on playing, as described in the following comment:

Teacher 2: They wanted to play that song all the time together, many hours. They had this tension in the class all the time, and students were stressed because of that. It was, like, we can do this together, and we are able to do this together, and there was this ease of doing.

These findings emphasize the relational resources of agency, such as trust between peers and a teacher, equality, and a musical environment that creates a sense of safety as such. According to our findings, emotions such as relief and ease take over from fear and insecurity, and they nourish the self-efficacy and agentic participation of the individuals. The findings discussed here suggest that the safety in joint music-making is musically and socially constructed through the experience of entrainment and takes shape as dialog that goes beyond words. These aspects of intersubjectivity through the experience of entrainment are resourcing an individual so they can develop their competence without the fear of making mistakes and establish trust for peers by acknowledging their efforts and supporting them when personally playing something incorrectly. Non-verbal abstract dialog creates opportunities to search for new ways of learning how to build trust and affirm social growth when it is challenging in the verbal world.

### Continuity

Continuity is the particular characteristic of the process in joint music-making, which describes the cumulative process toward and through entrainment and its connections to competence, self-efficacy, and motivation as it creates possibilities for participation. Both students and teachers described this continuity when they were relating the processes of joint music-making in their classes, and it was closely linked to interactions between the teacher and students, their relationships, and the way teachers facilitated and supported their students’ participation. In particular, a comment from one teacher described the nature of continuity and its affordances for the mentioned characteristics of agency:

Teacher 9: I see joint music-making playing as a puzzle. First, you give the easy pieces for everyone, and we start to slot them together where they belong. And when I see those easy pieces go to their own places, or if not, I can give those more difficult pieces to those students who managed to slot the easy pieces already.

Many teachers explained how they looped the song continuously so that every student got to come along and how they built up the song with their students by adding new layers, instruments, and rhythms on the fly and stabilizing the joint tempo. In the process, students could then choose their own ways of participation, as two teachers stated:

Teacher 8: We start with the simple things, and only after that do we add something new so that every student can participate at their own level.

Teacher 4: After the moment that everyone has had time to get to know the instrument and chords they are about to play, I start to accompany them with piano by looping the chord progression and say to them: “Come along and play those chords that you can and know,” and once I see frustrated students who would want it to succeed right away, I remind them, “Play the chord that you can. It is more than enough!” And I see them calming down when they succeed. And they realize it is enough in this moment now.

Students were also aware of the nature of the process, interaction, and continuity as they described the support they needed and got from their teacher. They explained how the teacher “gives instructions how to get started,” shows “chords from the screen to keep us together,” or “accompanies” their students to help them maintain the entrainment and to help them to find their own ways of participating, as in the following extracts from students:

Student 21: Teacher gives options for how to play more easily. “Try that one first” and then “After that, you can start to play more,” and it makes it much easier.

Student 2: We always do it first in an easy way, and we slowly fasten tempo. So everyone gets to come along.

The goal of the process seems to be the moment when the teacher no longer supports the entrainment, but students themselves carry the entrainment themselves. That means, from the agency point of view, that students have found their individual ways of participating in their environment and are competent enough to work safely and collectively with their peers, leaning on their peers’ support but bringing in their own efforts as well to that moment. Again, emotions related to pride, success, and joy are present in these moments, as a student and some of the teachers noted:

Student 2: If it succeeds well, we do not need the teacher to accompany us with any instrument.

Teacher 6: It is so nice when we can let go of the backing track and students can be, like, “Now WE played this!”

Teacher 5: I always aim at that moment when I am no longer needed and my students themselves can maintain and go through the song. When I can leave my instrument and the song just goes on, I always praise my students, how they are so skillful and how wonderful it is that they can do it on their own.

Teacher 3: The idea of using backing tracks is that they keep the whole thing together. But, at some time, you can let them go once the students can act independently enough that they can play the whole song through without any help.

Teacher 11: In some classes, students just say to me, “Teacher, you don’t have to show the chords to us any longer.” because they just know. I just say to them, “You see how you start to learn and perceive? I am no longer needed because you know and feel when it is time to change the chord”.

However, there were also teachers who were struggling with the resistance of the students:

Teacher 3: I am wondering how the students are experiencing a classmate being resistant, like if he just refuses, does not want to, or is not able to participate in joint music-making. Like if there is someone who fools around or discloses not knowing how or refuses to do something, which I experience quite often. Like what can I do? Is it about the skill, insecurity, or maybe a psychological problem why it is so hard for someone? Like, can we know why joint music-making works with some class and why not with some other class?

Teacher 11: I see it almost every day how a student does not even try, or maybe tries a bit but then gives up. This same thing appears within different classes as well with the same student, like how you are able to try. And how quickly you tend to give up when you feel something is challenging and do not learn it quickly.

The extracts above provide insights into the unambiguous side of agency when students might refuse, or even reject, the opportunities to become part of the group or refuse to participate in a way that the teacher desires. However, the findings of the study underscore the richness of the positive experiences the students have outlined, which teacher perspectives have affirmed, like the cumulative process of joint music-making, thus highlighting the relational resources of agency, such as interaction, guidance, and support needed from the teacher and pointing to the opportunities of participating. Through participatory resources of agency, such as opportunities and possibilities to choose, students can move freely toward and through entrainment and thereby stabilize their skills and strengthen their sense of competence and self-efficacy. However, the agency in the joint music-making may be constrained by synchronous collaborative action since the possibilities for individual action are limited. Thereby, the complexity of the aspects of continuity in joint music-making form a significant constraint on agency, suggesting temporality and the individual’s self-efficacy, which can create an unpleasant loop where an individual loses the freedom to be agentic and hence demonstrates resistant agency such as withdrawing from joint activity. The findings also emphasize the role of the teacher and their responsiveness and pedagogical sensitivity when guiding students through joint music-making.

## Discussion

This study provides a unique view into how agency and entrainment resource and constrain each other in joint music-making. In response to the first research question concerning the characteristics of agency present in the reported student and teacher experiences in joint music-making, four themes were identified that offer different lenses through which to view agency. Presence in joint music-making approaches agency as an embodied, active, and intentional way of being aware and sensing the shared emotions that are taking shape through musical and social acts in the here and now. Belonging, in turn, involves the agentic participation that is at the core of the ongoing musical and social interaction featured by perception and emotions. Safety indicates how joint music-making creates a non-verbal dialogical space for agency and a platform to affirm social growth, but also how perceiving musical elements through entrainment in joint music-making facilitates emotions of ease and relief and creates a sense of safety for an individual to confront and overcome their insecurity. Finally, Continuity clarifies the agentic process of an individual during joint music-making and how, through the opportunities and possibilities to choose, the way opens up to strengthen self-efficacy and competence.

Second, we investigated the ways in which agency and entrainment resource and constrain each other in joint music-making in the reported experiences of students and teachers. Our study suggests that music and joint music-making function as an invitation for individual agency, as joint synchronous musical action creates a potential environment and opportunity for participation by bringing individuals together in the present moment through entrainment. Musical entrainment becomes an intersubjective experience as individuals make their way to togetherness not only through the musical experience but also through relational and dialogical paths as they are responsive, respectful, and responsible toward others and where emotional encounters are taking shape through joint music-making. Our findings suggest that music produced in joint music-making functions as a form of feedback and a resource for an individual, as by perceiving the tightness or looseness of entrainment, as individuals and as a group, there is an immediate sense of togetherness, raising positive emotions that further enrich the sense of togetherness—we did this, and we sound good! In this feedback loop, awareness, anticipation, and reflection come together, reinforcing one another, fostering the self-efficacy of an individual, a sense of belonging, and confirming the intersubjective experience of joint music-making.

The findings of this study provide insights into the constitutive nature of the relationship among agency, entrainment, and intersubjectivity as they highlight the role of becoming aware of the present moment, emotions facilitating or limiting the process, musical and social togetherness, and the sense of safety, but also the complexity of aspects that may constrain agency and lead to resistance and withdrawal from the activity. The intersubjective experience of entrainment in joint music-making opens a dialogical space for relationships through music and body (Vass, 2019), for both students and teachers, which functions as a platform for agentic participation and the development of relational resource and social safety, which are central for agency (Juutilainen et al., 2018). Thus, this dialogical space can be regarded as a significant resource for agency. Particularly, as the experience of entrainment is often featured by positive affects, and as emotions are closely linked to our agency when considering what is worth pursuing, these emotions are featured by the experience of entrainment and resource agency in versatile ways. However, the temporal aspect of agency or the temporal ongoing process of the experience of entrainment shaped by emotions, in other words, the previous experiences of an individual and how an individual tends to give up when learning something new, seems to be either a remarkable resource or a constraint for agency. Moreover, the findings indicate that there is more to understand with regard to this phenomenon.

The findings of this study indicate how experienced music-related entrainment intertwines with intersubjectivity, as the findings emphasize the active feeling, sharing, and being collectively in the very present moment by means of music. Hence, playing together opens a unique space for dialogical relationships that are an important part of intersubjectivity (Vass, 2019). The findings support the understanding of intersubjectivity (Stern, 2004; Trondalen, 2016), the core of which is becoming aware of the collective moment and experiencing each other through unspoken interaction as the microprocesses lead the way. Additionally, the findings elaborate on how in joint music-making, this shared understanding is highly interactive and non-verbal (Phillips-Silver and Keller, 2012) and how it forms a base for one’s experiences of meaning, togetherness, possibilities for action, and subjectivity (Trondalen, 2016), which are also central to the human agency (Eteläpelto et al., 2013).

Through music and the embodied resonance in joint music-making, it is possible to sense and become aware of others, respond to and interact with them, and get the experience of both becoming visible and understood at an abstract level that goes beyond the verbal world. The emotionally safe climate has been recognized as fostering agency in previous studies of education (Eteläpelto et al., 2013; Ruohotie-Lyhty and Moate, 2016; Juutilainen et al., 2018; Jääskelä et al., 2020), but we believe that this study contributes to the discussion about the agency by emphasizing the importance of this unspoken way of interacting and relating to each other that forms a platform for building a significant resource of social trust. However, it is not only the non-verbal communication but also the particular role of music, the musical elements, and the experienced entrainment, such as perceived ongoing rhythm and dense musical texture (Turino, 2008; Ruud, 2020), that in joint music-making are essential to creating the sense of safety and giving rise to social contribution and equity. In educational studies, these are regarded as fundamental outcomes of agency (Vaughn, 2020). This might be the key in understanding how joint music-making can foster social growth (Pearce et al., 2015; Saarikallio, 2019), advance prosocial behavior (Kirschner and Tomasello, 2010; Mogan et al., 2017; Ilari et al., 2018), and bring people together regardless of their conflicting ways of thinking.

The findings of this study suggest that during joint music-making, the agency of an individual is constructed by and intertwined with the sense of belonging, which is achieved through perceiving being part of the musical and social entity and which is featured by strong affects. Previous studies link the experience of entrainment to positive feelings and the arousing of emotions because of the feeling of unity at different levels (Phillips-Silver and Keller, 2012), whereas in educational research, emotions are closely linked to the human agency as they lead the way to active participation (Eteläpelto et al., 2013; Slaby and Wüschner, 2014; Kirby, 2020; Ruud, 2020; Mameli et al., 2021). The results indicate that if there is a strong sense of belonging, there are likely also positive emotions and, therefore, a will to actively participate in and maintain something that exists that involves apparent manifestations of agency (Eteläpelto et al., 2013). Moreover, the results elucidate how the experience of entrainment increases the sense of belonging, as it is characterized by intersubjectivity as the affects that are taking shape through unspoken interaction among participants. In other words, the key finding of this study is in line with previous research that the quality of agency is highly intersubjective (Damşa et al., 2010; Vass and Littleton, 2010) in joint music-making, relationally constructed, non-verbally experienced, and shaped by affects and an environment where relational resources are an intrinsic prerequisite for active participation of an individual.

However, even though this study shows the richness of joint music-making and its affordances to the human agency, there are complexities and contradictions when it comes to agentic resistance and when there are students who might refuse to participate in such an experience. It is a challenge, particularly in such activity, that depends on the social and musical contribution of individuals to even exist in the here and now. This study emphasizes the meaning of holistic and affective experiences that the verbal world cannot reach. This might also be the answer to why an individual demonstrates resistance in joint music-making. As human agency (Emirbayer and Mische, 1998; Biesta and Tedder, 2007; Eteläpelto et al., 2013) is temporally constructed, there are always previous experiences of an individual that are realized in the present moment. Consequently, an individual does what they know from experience, and it might be challenging to them to see what opportunities and possibilities exist in the present. Moreover, if emotions are inextricably linked to experiences and, thus, to agency (Eteläpelto et al., 2013; Slaby and Wüschner, 2014; Ruud, 2020), it is no wonder that teachers are experiencing challenges with resistant students. Emotions have an effect on how we participate and give rise to the possibility of experience, and experiences evoke emotions that are true to ourselves. Thus, we as music educators should never underestimate the power of emotions that are, according to our findings, an intrinsic part of joint music practices, but we should pay extra attention to the risk of social isolation and keep in mind the delicate and emotional construct of agency.

This study also contributes to the discussion about the agency by bringing together both students’ and teachers’ experiences of agency in joint music-making that is characterized by entrainment and intersubjectivity. These experiences provide insights into how teachers and students’ agencies interact with each other in music classrooms, as resourcing or constraining, as together they create a human experience that makes them feel equal through collaborative musical act and entrainment. In other words, this study underscores the dyadic nature of agency in how teachers facilitate and continuously evaluate the opportunities for their students to participate in the musical environment and adapt their teaching based on the responses they get from their students. Conversely, the students similarly negotiate and consider their agency based on the possible opportunities offered by their relational and environmental reality. With regard to further research, we suggest that this encounter between the agencies of teachers and students should be further investigated.

### Limitations and future directions

The limitations of this study include the use of interviews of student and teacher participants without performative data that would provide insights into how the experiences and beliefs are enacted in practice. First, we are aware that some of the participants found it easier to share their experiences and thoughts than others, especially when it involved student participants. Yet, the data provided a broad picture of different individuals with diverse backgrounds and experiences. Second, a larger sample size, with more than one class of Grade 6 students and 11 teachers, would provide a more diverse picture of the experiences of students and teachers. Third, the study was conducted in Finland within an educational context that might differ from that of music education in schools in other countries. Future research could include students and teachers in different countries to see whether the findings are reflective of other cultures and contexts. Last, we are aware that our study did not measure how closely entrained our participants were, nor did we comment on the nature of entrainment. Moreover, if the data were explored from a different perspective, other considerations might come to light.

To reflect on the different levels of reflexivity of the study, it is worth acknowledging that this particular study belongs to a larger study examining different aspects of music education in Finnish primary education. The authors of this study have previously conducted two studies that have influenced the theoretical understanding of this study. Moreover, all the authors are not only researchers holding different specialties and supporting various epistemological and ontological positions but also teachers of different subjects, which influenced the way the study was conducted and how the data were collected, analyzed, and interpreted. However, data analysis included a double-coding process and discussions with co-authors to ensure the integrity of the results. Additionally, we have carefully described our interpretations of the data and linked these to existing theories throughout the findings to increase the reliability of the study.

The interviews provided insight into how, through music, our participants perceived themselves as being part of a musical and social entity, which made them experience strong positive emotions, such as enthusiasm when succeeding, which further made them willing to actively take responsibility in entering or maintaining joint music-making. Hence, based on our results, future research could investigate how the participants respond to someone not maintaining tempo and how it affects their sense of belonging. Nevertheless, we believe our study provides valuable insights into the relationship between agency and entrainment in joint music-making and shows that there is more to be studied.

### Implications

Based on the experiences of both students and teachers, it is clear how there are some human interactions such as joint music-making, which is characterized by entrainment and intersubjectivity, that cannot be replaced by providing verbal meanings in place of actual experience. Our study offers a holistic picture of the dialogical space of joint music-making where agency is resourced and constrained through the ongoing, affective process of interaction with music and other people in the here and now. We suggest that it is important to recognize the emotional side of agency, the inner experiences, and the importance of teacher sensitivity when guiding students through educational practices. These points should be addressed in both pre- and in-service teacher education since the holistic nature of joint music-making should be seriously considered as a means to better understand the complexity of agency in terms of both its richness and ambiguity. Moreover, as discussed earlier, the agency should not be understood only as a bare activity and eagerness to participate but also as passivity and withdrawal that often have social consequences. Based on our study, we suggest that the experience of entrainment is such an effective tool in fostering the agency of students and enhancing holistic learning but also that agency is a prerequisite for entrainment and intersubjectivity in joint music-making as it is dependable on the will and participation of an individual. Entrainment is not just an intrinsic part of music education; thus, we would encourage all educators to consider how education could better benefit from having music or experiences similar to those outlined in our study as part of the curriculum. After all, the most important goal of education is the development of human potential at both the subjective and collective levels.

## Conclusion

In the present study, we propose how entrainment and intersubjectivity are significant aspects of joint music-making based on the reported agentic experiences of students and teachers. Agency is an important aspect in enabling entrainment in joint music-making, and, in turn, the positive experience of entrainment feeds back to agency in versatile ways. It is notable that agency and entrainment are intertwined with intersubjectivity in the experiences of joint music-making in a way that they together form the whole experience. This means they can be analytically separated, but in practice, they complement each other. Furthermore, our study suggests how an experience of entrainment is conscious and which can be learned and aimed at, and how it feeds into the development of agency through the agentic processes.

## Data availability statement

The raw data supporting the conclusions of this article will be made available by the authors, without undue reservation.

## Ethics statement

Ethical review and approval was not required for the study on human participants in accordance with the local legislation and institutional requirements. Written informed consent to participate in this study was provided by the participants’ legal guardian/next of kin.

## Author contributions

ES was responsible for the original study design, data collection, material preparation, and data analysis and wrote the first draft of the manuscript. All authors contributed to the study’s conception and design, commented on previous versions of the manuscript, and read and approved the final version of this manuscript.

## Appendix

The questions in Appendices 1, 2 are constructed in a way that they would cover the individual (competence beliefs, self-efficacy, and intrinsic motivation), participatory (subject’s experience of the opportunities for active participation and influencing and making choices), and relational (emotional atmosphere and experiences of trust, support, and power relations) domains of student agency, as in the questions for students*:* “In your opinion, what is the nicest thing when your whole class plays and sings together?” and “Is there something that makes it not so nice?” Additionally, the important individual and collective aspects of musical agency, such as relationship with music, perceiving music, playing instruments, and collective coordinated action are present, as in the question for teachers: “In your opinion, what affects how a whole group of students starts to play together, and how is the joint music-making situation built?”

### Appendix 1

The questions used in the student interviews in this article are as follows:

Do you listen to music in your free time, and if so, what do you listen to? Is there an instrument that you especially enjoy playing, and why? Is there an instrument that you do not enjoy playing, and why? If you begin to play a new song with your class, what do you usually do, and how does the process go? When you begin to play a song with your class, what are the things encouraging you to participate? Are there things that make you not want to participate? In your opinion, what is the nicest thing when your whole class plays and sings together? Is there something that makes it not so nice? What does your teacher do when you start to play together as a class? How does your teacher teach you?

### Appendix 2

The questions used in the teacher interviews in this article are as follows:

What kind of experiences do you have of playing as a group in your personal life, and what kind of meanings do they have for you? In your opinion, what affects how an individual student participates in joint music-making? In your opinion, what affects how a whole group of students starts to play together, and how is the joint music-making situation built? How do you support an individual student and the whole group in the joint music-making situation as a teacher? Is there something concerning joint music-making we have forgotten to talk about in addition to what we have discussed already? Is there something you want to mention or talk about?
